# Computational Design of a Multi-Epitope Vaccine Against *Porphyromonas gingivalis*

**DOI:** 10.3389/fimmu.2022.806825

**Published:** 2022-02-18

**Authors:** Bilal Shaker, Sajjad Ahmad, Junhao Shen, Hyung Wook Kim, Dokyun Na

**Affiliations:** ^1^ Department of Biomedical Engineering, Chung-Ang University, Seoul, South Korea; ^2^ Department of Health and Biological Sciences, Abasyn University, Peshawar, Pakistan; ^3^ College of Life Sciences, Sejong University, Seoul, South Korea

**Keywords:** *Porphyromonas gingivalis*, immunoinformatics, molecular docking, molecular dynamics simulations, epitopes, vaccines

## Abstract

*Porphyromonas gingivalis* is a Gram-negative pathogenic bacterium associated with chronic periodontitis. The development of a chimeric peptide-based vaccine targeting this pathogen could be highly beneficial in preventing oral bone loss as well as other severe gum diseases. We applied a computational framework to design a multi-epitope-based vaccine candidate against *P. gingivalis*. The vaccine comprises epitopes from subunit proteins prioritized from the *P. gingivalis* reference strain (*P. gingivalis* ATCC 33277) using several reported vaccine properties. Protein-based subunit vaccines were prioritized through genomics techniques. Epitope prediction was performed using immunoinformatic servers and tools. Molecular modeling approaches were used to build a putative three-dimensional structure of the vaccine to understand its interactions with host immune cells through biophysical techniques such as molecular docking simulation studies and binding free energy methods. Genome subtraction identified 18 vaccine targets: six outer-membrane, nine cytoplasmic membrane-, one periplasmic, and two extracellular proteins. These proteins passed different vaccine checks required for the successful development of a vaccine candidate. The shortlisted proteins were subjected to immunoinformatic analysis to map B-cell derived T-cell epitopes, and antigenic, water-soluble, non-toxic, and good binders of DRB1*0101 were selected. The epitopes were then modeled into a multi-epitope peptide vaccine construct (linked epitopes plus adjuvant) to enhance immunogenicity and effectively engage both innate and adaptive immunity. Further, the molecular docking approach was used to determine the binding conformation of the vaccine to TLR2 innate immune receptor. Molecular dynamics simulations and binding free energy calculations of the vaccine–TLR2 complex were performed to highlight key intermolecular binding energies. Findings of this study will be useful for vaccine developers to design an effective vaccine for chronic periodontitis pathogens, specifically *P. gingivalis*.

## Introduction

*Porphyromonas gingivalis* is a rod-shaped, Gram-negative oral bacterium asserted as a pivotal pathogen in the growth of chronic periodontitis (1). Periodontitis is caused by the inflammation of periodontal tissues and may result in tooth loss (2). *P. gingivalis* is detected in 85% of periodontitis sites, is rarely detected in healthy patients (3), and can modulate periodontal protective mechanisms (4–6). *P. gingivalis* may exacerbate cognitive impairments in patients with Alzheimer’s disease; oral infection by *P. gingivalis* led to the activation of a complement pathway in the mouse brain tissue (7), and periodontitis-induced Alzheimer’s disease model mice showed impaired cognitive function compared to non-induced mice (8).

Vaccines are biological products that stimulate and induce acquired immunity against a particular pathogen and are the most efficient and cost-effective method to prevent infectious diseases (9, 10). There were several attempts to develop attenuated vaccines against *P. gingivalis*, but satisfactory levels of protection were not observed. For example, Polak et al. (11) observed humoral immune responses elicited against *P. gingivalis* in the immunized mice, but the vaccine failed to prevent periodontitis. Leone et al. (12) demonstrated that immunized mice against *P. gingivalis* showed increased inflammation and fibroblast apoptosis, and exacerbated bone loss through the up-regulation of innate immune response. Immunization with specific proteins such as fimbriae and RgpA from *P. gingivalis*, instead of whole bacterial materials, was found to be more protective against periodontitis and oral bone loss (13–22). These results emphasize the need for identifying proteins responsible for boosted vaccination. Since the key components inducing immune response are the epitopes included in proteins, there is a need to identify responsible antigenic sequences from proteins and optimize the sequences for enhanced vaccines (22).

Over the past decades, the vaccine field has been revolutionized owing to the advances in structural biology, genomics, computational biology, and biotechnology (23, 24). A new technology, named reverse vaccinology, allows for identifying virulence factors of pathogens responsible for unmet diseases from their genomic sequences, discovering antigenic sequences, and optimizing the sequences *via* computational methods (25). The reverse vaccinology approach was first applied to *Neisseria meningitidis* serogroup B for which no broad-spectrum vaccines were available due to the variations in their outer-membrane proteins and the potential cross-reactivity of their capsular polysaccharide with human tissues (26). After this successful attempt, reverse vaccinology has been accepted as an effective method for vaccine discovery.

In this study, we aimed to develop a vaccine to provide protective immunity against *P. gingivalis* that causes chronic periodontitis in humans, and is likely correlated with the severity of Alzheimer’s disease symptoms. We adopted a pan-genomic reverse vaccinology strategy to shed light on the core proteins shared across all strains of the pathogen and unique proteins present in selected strains, which allows for the development of both broad- and narrow-spectrum vaccines (27). Subtractive proteomic analysis further discarded the proteins of the pathogen homologous to human proteins. After the prioritization of potential vaccine target proteins, multiple epitopes that can interact with major histocompatibility complex (MHC) class I and II proteins were predicted from the target proteins to maximize both B- and T-cell immune responses (28). Subsequently, world human population coverage by predicted MHC class I and II was also analyzed (29). To boost the immune system, a multi-epitope chimeric protein was designed to ensure sufficient solubility and stability *via* molecular dynamics simulations (30). The designed vaccine protein contained the predicted epitopes tagged using a flexible linker, and Pam3CSK4 chain C (PDB ID: 2Z7X) was used as a vaccine adjuvant (31–33) to boost the antigenicity of predicted epitopes and guide epitope recognition and processing. The computational methodology developed in this study will facilitate the development of reliable and effective vaccines to fight against pathogens. In addition, the designed vaccine construct may provide a new method to treat chronic periodontitis and alleviate the symptoms of Alzheimer’s disease.

## Material and Methods

### Antigenic Protein Selection From Proteome of *P. gingivalis*

The complete proteome of *P. gingivalis* (ATCC 33277) was obtained from the National Center for Biotechnology Information database (34). To shortlist proteins for vaccine construction, the whole proteome of the pathogen was subjected to a subtractive pipeline (35). First, duplicate or very similar proteins were discarded using the Cluster Database at High Identity with Tolerance (CD-HIT) suite with a sequence identity cut-off of 0.9 (36). Second, proteins predicted to be located within the cytoplasm using the PSORTb subcellular localization prediction tool (37) were discarded because cytoplasmic proteins cannot be recognized by host immune systems; only proteins in the outer or periplasmic membranes play important roles in the attachment, infection, and survival of pathogens (38). Third, the proteins homologous to human proteins were discarded to avoid autoimmune responses using the protein Basic Local Alignment Search Tool (BLASTp) with a cutoff E-value of 10^-4^ (39, 40). The remaining proteins were subjected to the subsequent processes including virulency-, molecular weight-, and antigenicity prediction.

Virulent proteins are promising candidates for vaccine design because they play an important role in pathogenicity, interact with host pathways, and thus are conserved across the strains of pathogens (41–43). Therefore, they are major targets for vaccines against multiple strains. Of the selected proteins in previous processes, those similar to known virulence factors deposited in the Virulence Factor Database (44) were selected. The search was carried out by using BLASTp with a sequence identity >30% and a bit score >100.

The potential antigenicity of the selected virulent proteins that were non-homologous to human proteins and were not located within the cytoplasm was predicted by using VaxiJen (45). The proteins with a predicted antigenicity >0.4 (default cutoff of VaxiJen) and a molecular mass <110 kDa were selected (42, 43) and used as target proteins for epitope search.

### Epitope Prediction Within Selected Antigenic Proteins

Potential epitopes to stimulate cell-mediated immune responses were predicted on the selected proteins from the previous processes by using NetCTL with a default setting (46). NetCTL predicted cytotoxic T lymphocyte (CTL) epitopes capable of interacting with class I MHC proteins and thereby stimulating the activation of CTLs based on the binding affinity between class I MHC proteins and epitopes, proteasomal C-terminal cleavage, and transporter associated with antigen processing (TAP) transport efficiency (46). The predicted CTL epitopes could elicit cell-mediated immunity to inhibit the development of the pathogen and induce the generation of memory T cells to prevent future infection (47).

B-cell epitopes are specific peptides within antigens recognized by B-cell receptors and antibodies produced from the activated B cells. Stimulation of humoral immune response is also important because it can clear pathogens by inducing an antibody-mediated immune response. Linear B-cell epitopes within the selected proteins were predicted using the ABCpred server (48).

Full activation of stimulated immune cells (T and B cells) requires additional stimulation from helper T lymphocytes (HTL) to avoid autoimmune responses. HTL recognizes epitopes loaded onto class II MHC proteins in B-cell membranes and other antigen-presenting cells. The HTL epitopes were predicted by using an online tool served by the Immune Epitope Database (IEDB) (49). This tool ranked predicted epitopes based on a percentile score calculated by comparing the scores generated using three methods (SMM-align, Combinatorial library, and Sturniolo) with the scores of five million random 15-mer peptides generated from the proteins in the UniProt database (50, 51).

### Interferon-Gamma Epitope Prediction

The IFNepitope webserver was used to predict the MHC-II (HTL) epitopes that can induce interferon-gamma with an 82% accuracy (52). The server uses machine learning-based models including motif-based model, support vector machine-based model, and hybrid approach (motif and support vector machine) to predict the interferon-producing property of epitopes and assigns support vector machine scores to each input epitope.

### CTL and HTL Epitope Screening

Predicted CTL and HTL epitopes were further shortlisted by applying multiple filters including toxicity, antigenicity, MHC binding affinity, and solubility. Online web servers VaxiJen (45), ToxinPred (53), MHCPred (54), and peptide solubility calculator (https://pepcalc.com/peptide-solubility-calculator.php) were used to predict the toxicity, antigenicity, MHC binding affinity, and water solubility of each epitope, respectively. Epitopes predicted as non-toxic, antigenic, and water-soluble positive binders were selected for vaccine design.

### Population Coverage by CTL and HTL Epitopes

Human leukocyte antigen (HLA) pattern varies among ethnic groups and geographical areas. Thus, the estimation of population coverage by the predicted CTL and HTL epitopes is important to design an effective vaccine. The population coverage tool of IEDB (http://tools.iedb.org/population/) was used to estimate the world human population coverage by the predicted CTL and HTL epitopes (49).

### Multi-Epitope Peptide Vaccine Construction

In this step, shortlisted epitopes were linked using two linkers, AAY and GPGPG (55). The AAY linker was used to fuse CTL epitopes, whereas the GPGPG linker was used to fuse HTL epitopes. These linkers help separate the epitopes to avoid junctional epitope (neo-epitopes) formation and improve epitope presentation (56–59). Furthermore, Pam3CSK4 chain C (PDB ID: 2Z7X) was used as a vaccine adjuvant to increase the immunogenic property of the vaccine construct (33). Adjuvant was linked at the N-terminus of the vaccine construct by using the EAAAK linker (60).

### Antigenicity and Allergenicity Prediction of Vaccine Construct

VaxiJen v2.0 (45) was used to predict the antigenicity of the vaccine construct. This server generates the antigenic score of the query sequence with the precision of 70–89% by using an alignment-independent algorithm. The non-allergenic behavior of the vaccine was predicted using the AllerCatPro server (61), which predicts the allergenic potential of peptides based on the three-dimensional (3D) structural similarity and compares their amino acid sequence with a dataset of known allergen proteins. The dataset used in this server was derived from the union of the major databases such as Food Allergy Research and Resources Program, Comprehensive Protein Allergen Resource, WHO/International Union of Immunological Societies, UniProtKB, and Allergome (62).

### Physicochemical Properties, Structure Prediction, Refinement, and Validation

ProtParam (63) was employed to determine the physicochemical properties of the vaccine construct, namely molecular weight, amino acid composition, theoretical isoelectric point value (PI), stability index, *in vitro* and *in vivo* half-life estimation, and GRAVY (grand average of hydropathicity). Further, the vaccine construct sequence was subjected to the PDBSUM server (64) for secondary structure prediction. The Robetta server (65) was used to generate the 3D structure of the vaccine construct. Robetta performs comparative modeling if there is a suitable template in a database; if there is no template, then the *ab initio* structure prediction method is employed. The fragment-guided molecular dynamics (66) algorithm was used for structure refinement at the atomic level. Subsequently, the refined predicted model was validated by employing VARIFY 3D (67), ProSA-web server (68), and the Ramachandran plot (69).

### Protein Disulfide Engineering

Protein model stability can be enhanced by adopting a disulfide bond formation approach. Disulfide bonds strengthen the geometric conformation of the protein structure and stabilize the structure. The protein structure was subjected to an online server DbD2 (70) for disulfide engineering. This web server can detect residue pairs with the ability to form disulfide bonds if the individual amino acid mutated to cysteine.

### Molecular Docking With TLR2

Molecular docking is an *in silico* technique to evaluate the binding affinity between ligand and receptor molecules (71). Human toll-like receptor 2 (TLR2) was used in this study to determine the binding affinity between the vaccine construct and human TLRs. The 3D structure of TLR2 was retrieved from the Protein Data Bank (PDB) (72) (PDB ID: 5D3I). The PatchDock server (73) was used to perform molecular docking, and the 3D structure of human TLR2 was uploaded as a receptor and vaccine structure as a ligand. Subsequently, the FireDock server (74) was used to refine the docked complex obtained from the PatchDock server.

### Molecular Dynamics Simulation

Molecular dynamics simulation (75) was performed to understand the stability and protein-protein interactions at the atomic level to support the predictions. Amber v.20 (76) software was used to run a 100 ns simulation of the selected complex. The LeaP module was utilized to add hydrogen atoms to the docked complex of the vaccine and the receptor (77). Then counter ions (Na^+^) were introduced to neutralize the simulated system. Subsequently, a truncated octahedral box of the TIP3P water model with buffer was submerged with the neutralized system (78). The solvated system was minimized first for the 1500 steps of the steepest descent method and then for the 1000 steps of the conjugated gradient method by using the ff14SB force field (79). In the next step, the system was heated up to 100 ps, reaching an equilibrium after 100 ps. The SHAKE algorithm (80) was applied to constrain bonds involving hydrogen atoms. A molecular dynamics simulation run was accomplished for 100 ns to determine the dynamics of the complex and evaluate the docked conformation and stability of the ligand. The CPPTRAJ module in Amber was used to perform trajectory analysis (81).

### MMPBSA/MMGBSA Binding Free Energy of the Complex

Two efficient energy calculation methods were used to estimate the binding free energy, namely MMPBSA (Molecular Mechanics Poisson-Boltzmann Surface Area) and MMGBSA (Molecular Mechanics-Generalized Born Surface Area), embedded in the MMPBSA.py module of Amber (82). The net energy of the system was calculated using equation (1).


(1) ΔGBinding=ΔGcomplex−ΔGReceptor−ΔGInhibitor

The terms in equation (1) include several energy calculations including van der Waals energy, internal energy from molecular mechanics, electrostatic energy, and polar and non-polar contribution towards solvation energy.

## Results and Discussion

**Figure 1:**
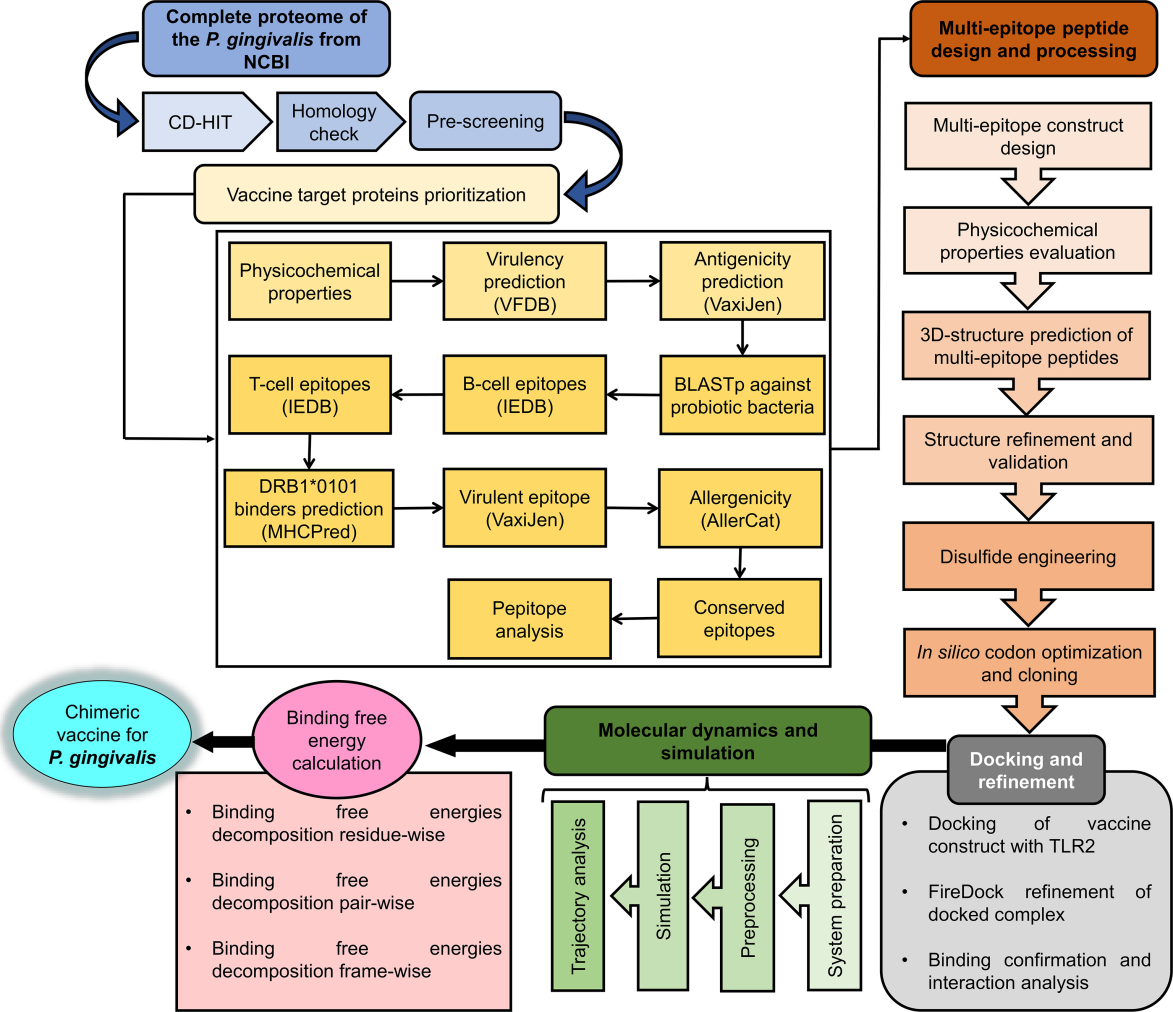
Stepwise workflow designed for *in silico* vaccine design against *P. gingivalis*.

**Figure 2:**
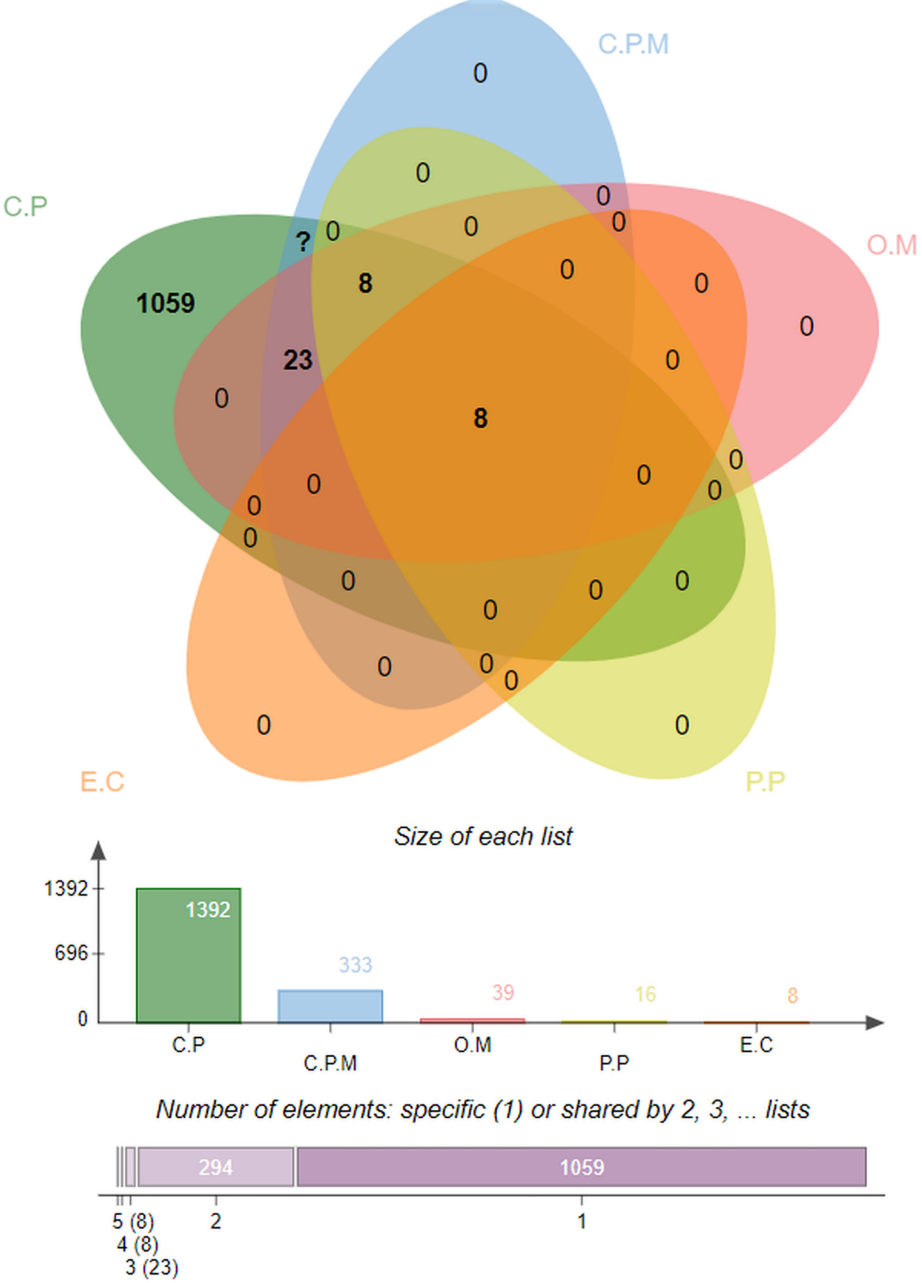
Percent-wise distribution of proteins within the cell, where C.P denotes Cytoplasmic, C.P.M is for Cytoplasmic-membrane, O.M represents Outer-membrane, P.P used for Periplasmic, and E.C is for Extracellular.

Several advanced sequencing techniques are being used for microorganism genome sequencing and thus hundreds of thousands of complete genomes are now available in sequence databases allowing the prediction of vaccine and drug targets (83, 84). In this study, immunoinformatics and subtractive proteomics techniques were applied to identify suitable proteins and design multiple epitope-based vaccines against *P. gingivalis* infection (85). Schematic diagram of the complete methodology to design a multi-epitope peptide vaccine against *P. gingivalis* is displayed in **Figure 1**. The complete proteome of *P. gingivalis* strain ATCC33277, comprising 1835 proteins, was collected from the National Center for Biotechnology Information (86). In the first step, the complete protein sequences of the pathogen were compared with each other to remove those that share the sequence identity of the set cut-off as stated in the methodology. For this purpose, the CD-HIT tool (87) was employed to identify 1788 non-paralogous and non-redundant proteins at a 90% identity threshold. These unique sequences are considered excellent starting materials in the vaccine and drug discovery process (88). These non-paralogous proteins were subjected to the PSORTb server (37) to predict the subcellular localization of proteins. The server categorized the proteins based on their location within the cell as 333 cytoplasmic-membrane, 8 extracellular, 39 outer-membrane, 16 periplasmic, and 1392 cytoplasmic proteins (**Figure 2**). Subsequently, cytoplasmic proteins were discarded because they are inaccessible to the host immune system and least suitable for vaccine design (38). Additionally, these proteins are enzymatic in nature to catalyze cellular processes and possess hydrophobic pockets for substrate binding (89). On the contrary, secretome/surfome/periplasmic proteins are surface-exposed, and host immune response can recognize antigenic epitopes of such proteins more efficiently for producing prompt and targeted immune reactions (90).

### Virulent Protein Analysis

Virulent proteins are crucial for pathogen survival and cause infection; they help the pathogen in bypassing the host immunity, invading host cells, dissemination, intracellular survival, and proliferation, which make them suitable for vaccine design (91). The BLASTp tool of the Virulence Factor Database was used to identify virulent proteins. Virulent proteins are explored often as potent vaccine targets and are responsible for infection establishment and pathological conditions (43). Proteins with identity scores >30% and a bit score >100% were considered virulent and selected for further evaluation. As a result, 30 cytoplasmic-membrane, 2 extracellular, 6 outer-membrane, and 4 periplasmic proteins were determined as virulent.

### Antigenic and Small-Size Protein Prediction

Different physicochemical properties (antigenicity and size) of the pooled virulent proteins were further evaluated to guide the selection of suitable vaccine candidates for experimental evaluation that can be readily used in vaccine development (40, 42). VaxiJen server (45) predicted the antigenicity of the virulent proteins. Proteins predicted as antigenic were selected, and subsequently, the ProtParam tool (63) was used to calculate the molecular weight of each antigenic protein. Since proteins with smaller molecular weight are easier to isolate and purify for structural and functional studies (41), proteins with molecular weight lower than 110 kDa were selected. Further, selected proteins were submitted to the BLASTp (92) tool of the National Center for Biotechnology Information to search for *P. gingivalis* for human homologs at the 10^-4^ cut-off value. Homolog proteins (sequence identity >30%) were discarded subsequently. Selecting human homologs could initiate the autoimmune response, negatively impacting the host’s healthy cells and tissues (93). Nine cytoplasmic-membrane, two extracellular, one periplasmic, and six outer-membrane proteins were antigenic with molecular weights lower than 110 kDa, and they were not homologous to the human proteome. A list of selected proteins is shown in **Table 1**.

**Table 1:** Selected proteins from *P. gingivalis* proteome for multiple epitope prediction.

Protein Localization	Protein ID	VaxiJen prediction	Sequence Identity with the human proteome	ProtParam (MW)
Cytoplasmic-membrane	WP_004585352.1	Antigenic	Not found	25 kDa
	WP_005875177.1	Antigenic	Not found	15 kDa
	WP_012457680.1	Antigenic	27%	67 kDa
	WP_012457906.1	Antigenic	29%	65 kDa
	WP_012458016.1	Antigenic	Not found	94 kDa
	WP_012458551.1	Antigenic	Not found	80 kDa
	WP_012458559.1	Antigenic	Not found	49 kDa
	WP_012458596.1	Antigenic	Not found	54 kDa
	WP_043876323.1	Antigenic	Not found	58 kDa
Extracellular	WP_012458254.1	Antigenic	Not found	110 kDa
	WP_012458360.1	Antigenic	Not found	31 kDa
Outer membrane	WP_004583707.1	Antigenic	Not found	22 kDa
	WP_012457596.1	Antigenic	Not found	73 kDa
	WP_012457732.1	Antigenic	Not found	42 kDa
	WP_012457733.1	Antigenic	Not found	43 kDa
	WP_012458162.1	Antigenic	Not found	74 kDa
	WP_039417044.1	Antigenic	Not found	50 kDa
Periplasmic	WP_012457845.1	Antigenic	Not found	45 kDa

### MHC-I and MHC-II Epitope Prediction

MHC-1 molecules are expressed on the cell surface of all nucleated cells. They present peptide fragments derived from intracellular proteins and play an important role in alerting the immune system against virally infected cells (94). NetCTL 1.2 server (46) was used to predict the MHC-I binding epitopes (9-mer) of each protein; 150 MHC-1 epitopes for cytoplasmic membrane, 41 epitopes for extracellular, 93 epitopes for outer-membrane, and 12 epitopes for periplasmic proteins were collected based on their high binding affinity. The IEDB MHC-II server (49) was used to predict MHC-II binding epitopes (15-mer) against a reference set of seven HLAs. MHC-II molecules are central to effective adaptive immune response and expressed on specialized antigen-presenting cells like B cells, dendritic cells, thymic epithelial cells, and monocytes (95). The top ten MHC-II binding epitopes with low percentile rank were retrieved from each protein. Both MHC-I and MHC-II epitopes were further subjected to a screening pipeline to determine suitable epitope candidates for a potent vaccine construct.

### Screening of MHC-I Epitopes

Antigenic, toxic, and water-soluble MHC-I epitopes were predicted using the VaxiJen server (45), ToxinPred (53), and peptide solubility calculator (https://pepcalc.com/peptide-solubility-calculator.php), respectively. Subsequently, half-maximal inhibitory concentration (IC50) values of MHC-I epitopes binding to HLA DRB1*0101 were predicted using the MHCPred server (54), and epitopes with IC50 <100 nm were selected.

Collectively, 16 MHC-I epitopes were selected for an effective vaccine construct based on their antigenicity, non-toxicity, water solubility, and IC50 binding with the receptor (**Table 2**). The IFNepitope server (52) was also used to search for interferon-gamma inducing epitopes. Positive binding epitopes were predicted and shortlisted for constructing the vaccine.

**Table 2:** Selected MHC-I epitopes for vaccine construct.

CTL-Epitopes	MHC binding affinity	VaxiJen	ToxinPred	IFN-epitope	MHCPred	Solubility
RMEVETLLY	0.7032	Antigen (0.47)	Non-Toxin (-1.26)	POSITIVE (0.463)	11.59	Good water solubility
HIEQQEQSY	0.3025	Antigen (0.74)	Non-Toxin (-1.14)	POSITIVE (0.095)	20.75	Good water solubility
DSDADAHIL	0.1990	Antigen (0.93)	Non-Toxin (-1.05)	POSITIVE (0.442)	6.67	Good water solubility
PTHPDHKAY	0.1826	Antigen (0.49)	Non-Toxin (-0.90)	POSITIVE (0.004)	17.34	Good water solubility
ETDEAYSYA	0.2099	Antigen (0.42)	Non-Toxin (-1.07)	POSITIVE (0.077)	67.61	Good water solubility
RLDIEVLLY	0.7684	Antigen (1.59)	Non-Toxin (-1.15)	POSITIVE (0.328)	7.11	Good water solubility
SSIGDVDVY	0.1774	Antigen (0.85)	Non-Toxin (-0.89)	POSITIVE (0.459)	39.54	Good water solubility
KSDGLLLQL	0.1498	Antigen (0.97)	Non-Toxin (-1.05)	POSITIVE (0.135)	2.94	Good water solubility
RLNSHWNEY	0.2847	Antigen (0.44)	Non-Toxin (-0.76)	POSITIVE (0.089)	24.5	Good water solubility
SITGKKISY	0.1109	Antigen (1.23)	Non-Toxin (-0.49)	POSITIVE (0.040)	6.9	Good water solubility
SANRRVEVY	0.1624	Antigen (1.04)	Non-Toxin (-1.29)	POSITIVE (0.123)	51.64	Good water solubility
RLDIENHFY	0.7342	Antigen (0.78)	Non-Toxin (-1.22)	POSITIVE (0.148)	79.07	Good water solubility
HSENANGEK	0.2454	Antigen (2.61)	Non-Toxin (-0.74)	POSITIVE (0.105)	523.6	Good water solubility
PSMSASGDY	0.3378	Antigen (1.12)	Non-Toxin (-0.99)	POSITIVE (0.042)	28.64	Good water solubility
SADDFGLAV	0.3207	Antigen (0.92)	Non-Toxin (-1.52)	POSITIVE (0.097)	14.45	Good water solubility
ETEAKAKEY	0.5234	Antigen (1.64)	Non-Toxin (-0.35)	POSITIVE (0.560)	5.75	Good water solubility

### Screening of MHC-II Epitopes

Predicted MHC-II epitopes were also introduced to the screening pipeline, and antigenic, non-toxic, positive interferon inducer, water-soluble, and low percentile-ranked epitopes were selected for vaccine design. Eleven selected 15-mer MHC-II epitopes are listed in **Table 3**.

**Table 3:** Selected potent MHC-II epitope candidates.

Peptides	VaxiJen prediction	ToxinPhred	IFN-epitope	Solubility	Percentile rank
RKGKIRTYINLLLTM	0.4687 (Probable ANTIGEN)	Non-Toxin (-0.30)	POSITIVE (0.406)	Good water solubility.	0.36
NRKGKIRTYINLLLT	0.5410 (Probable ANTIGEN)	Non-Toxin (-0.51)	POSITIVE (0.396)	Good water solubility.	0.42
QSGVQYRADKSYILA	0.5094 (Probable ANTIGEN)	Non-Toxin (-0.86)	POSITIVE (0.128)	Good water solubility.	0.02
SQSGVQYRADKSYIL	0.6590 (Probable ANTIGEN)	Non-Toxin (-0.79)	POSITIVE (0.023)	Good water solubility.	0.04
KNYRLASNFSTRFLS	0.6081 (Probable ANTIGEN)	Non-Toxin (-1.15)	POSITIVE (0.023)	Good water solubility.	0.12
TAKDPFRVSASARYD	0.9471 (Probable ANTIGEN)	Non-Toxin (-1.14)	POSITIVE (0.843)	Good water solubility.	0.19
NVVYFRINSAKIDRN	1.1461 (Probable ANTIGEN)	Non-Toxin (-1.32)	POSITIVE (0.048)	Good water solubility.	0.04
DNVVYFRINSAKIDR	0.8580 (Probable ANTIGEN)	Non-Toxin (-1.28)	POSITIVE (0.261)	Good water solubility.	0.05
LIRILTDNPDIRIEL	1.3259 (Probable ANTIGEN)	Non-Toxin (-1.07)	POSITIVE (0.016)	Good water solubility.	0.10
ELIRILTDNPDIRIE	1.0683 (Probable ANTIGEN)	Non-Toxin (-1.07)	POSITIVE (0.154)	Good water solubility.	0.12
DELIRILTDNPDIRI	0.7896 (Probable ANTIGEN)	Non-Toxin (-0.98)	POSITIVE (0.235)	Good water solubility.	0.26

### Population Coverage Analysis

The cumulative percentage of population coverage was estimated using the predicted epitopes in the vaccine construct in highly populated countries of South Asia, East Asia, Europe, and North America (**Supplementary Table 1**). The result indicates that the multi-epitope strategy could cover 99.93% of the human population: specifically, around 99% for European and North American, and 40-60% for Asian and African. The high coverage in European and American population and low coverage in Asian and African population represent that our vaccine construct may work better in European and American population. This might be due to biased HLA information in IEDB: when IEDB HLA dataset was analyzed, about half of the HLA dataset in IEBD was from European and American.

### Vaccine Construction

**Figure 3:**
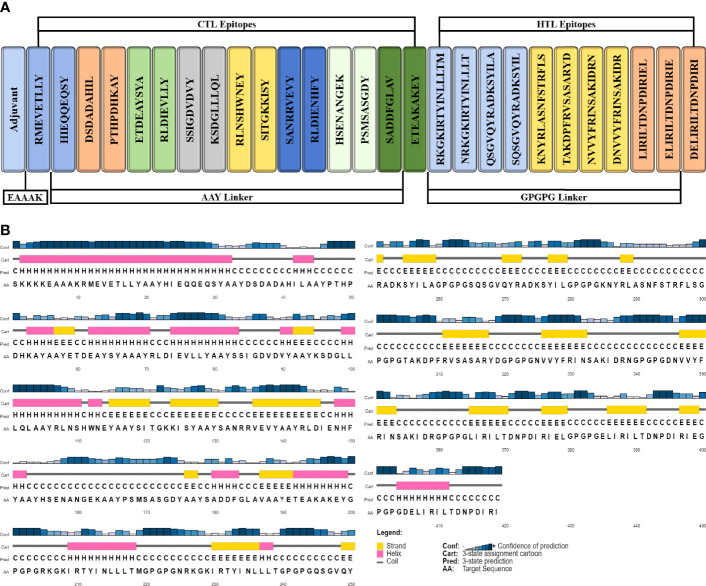
Schematic illustration of 419-amino-acid-long vaccine construct sequence. **(A)** Representation of adjuvant and epitope arrangement in vaccine construct and **(B)** 2D structure prediction of vaccine construct.

Organism- and peptide-based vaccines can successfully reduce the mortality and morbidity caused by infectious diseases (96). Peptide-based vaccines offer an attractive alternative to organism-based vaccines as they induce a specific immune response, are easy to synthesize and use in clinical settings, are cost-effective, minimize the risk of antigen-induced anaphylaxis, and are flexible in changing antigens (97). Nevertheless, during isolation, peptides are weakly immunogenic and require proper adjuvating (96). In this study, 16 CTL epitopes and 11 HTL epitopes were used to construct a vaccine sequence. The AAY and GPGPG linkers were used to fuse MHC-I and MHC-II epitopes, respectively. As TLR2 expression increases during *P. gingivalis* infection, TLR2 agonist Pam3CSK4 chain c (PDB ID; 2Z7X) was linked to the N-terminal of the vaccine construct using the EAAAK linker. The final vaccine construct composed of 419 residues containing both T-cell and B-cell epitopes were designed (**Figure 3A**). The vaccine construct is approximately 45.5 kDa. The construct can be easily purified by fusing with a short 6×histidine peptide or a fusion agent such as glutathione-S-transferase or maltose-binding protein. Even the fusion with the fusion agent (approximately 20-30 kDa), the recombinant protein would be easily produced and purified from *E. coli via* conventional protein expression techniques (98–100).

### B-Cell Epitope Prediction

The acquired/adaptive/specific immunity responses of the immune system are highly specialized and systemic in eradicating pathogens from the body or halting their growth (101). B-cells are major lymphocytes of adaptive immunity and are involved in generating humoral and cell-mediated immunity against specific and unwanted invader pathogens (101). In this study, the ABCpred server (48) predicted 14 protein sequence-based linear B-cell epitopes (20-mer) with a 0.8+ score (**Table 4** and **Supplementary Figure 1**). The DiscoTop2.0 server (102) predicted 202 discontinuous B-cell epitopes from the protein structure (**Supplementary Table 2**).

**Table 4:** Predicted linear B-cell epitopes.

Linear B-cell epitopes		
Sequence	Start position	Score
PGELIRILTDNPDIRIEGPG	383	0.95
GLIRILTDNPDIRIELGPGP	364	0.92
DNPDIRIEGPGPGDELIRIL	392	0.87
NSAKIDRNGPGPGDNVVYFR	332	0.86
PGPGTAKDPFRVSASARYDG	301	0.86
NPDIRIELGPGPGELIRILT	372	0.83
GSQSGVQYRADKSYILGPGP	264	0.83
LAAYPTHPDHKAYAAYETDE	43	0.82
DFGLAVAAYETEAKAKEYGP	182	0.82
SENANGEKAAYPSMSASGDY	156	0.82
NGPGPGDNVVYFRINSAKID	339	0.81
ARYDGPGPGNVVYFRINSAK	316	0.81
YAAYDSDADAHILAAYPTHP	31	0.81
QSGVQYRADKSYILAGPGPG	245	0.81

### Antigenicity and Allergenicity Prediction of Vaccine Construct

VaxiJen (45) and AllerCatPro (61) servers were used to predict antigenicity and allergenicity of the vaccine construct, respectively. The VaxiJen server calculated an antigenic score of 0.89 for the vaccine construct at 0.4 thresholds designating the vaccine potential to trigger host immune response. AllerCatPro (61) showed no evidence (no sequence and structural similarity with known allergens) for vaccine allergenicity prediction.

### Prediction of Physicochemical Properties

The ProtParam tool (63) was employed to compute the physicochemical properties of the vaccine construct. The molecular weight was 45.5 kDa and the theoretical PI was 7.69, whereas the vaccine construct was classified as stable with an instability index of 29.95. Furthermore, the estimated half-life (*in vivo*) of the vaccine construct in yeast and *Escherichia coli* was >20 and >10 h, respectively. The aliphatic index indicates the thermostability of the vaccine and was calculated as 77.49, whereas the GRAVY was calculated as -0.488.

### Secondary Structure Prediction

The secondary structure was generated by using the PSIPRED workbench (103). The vaccine structure was composed of 26.2% alpha helices, 23.6% beta strands, and 50.1% coils (**Figure 3B**).

### 3D Structure Prediction and Validation

**Figure 4:**
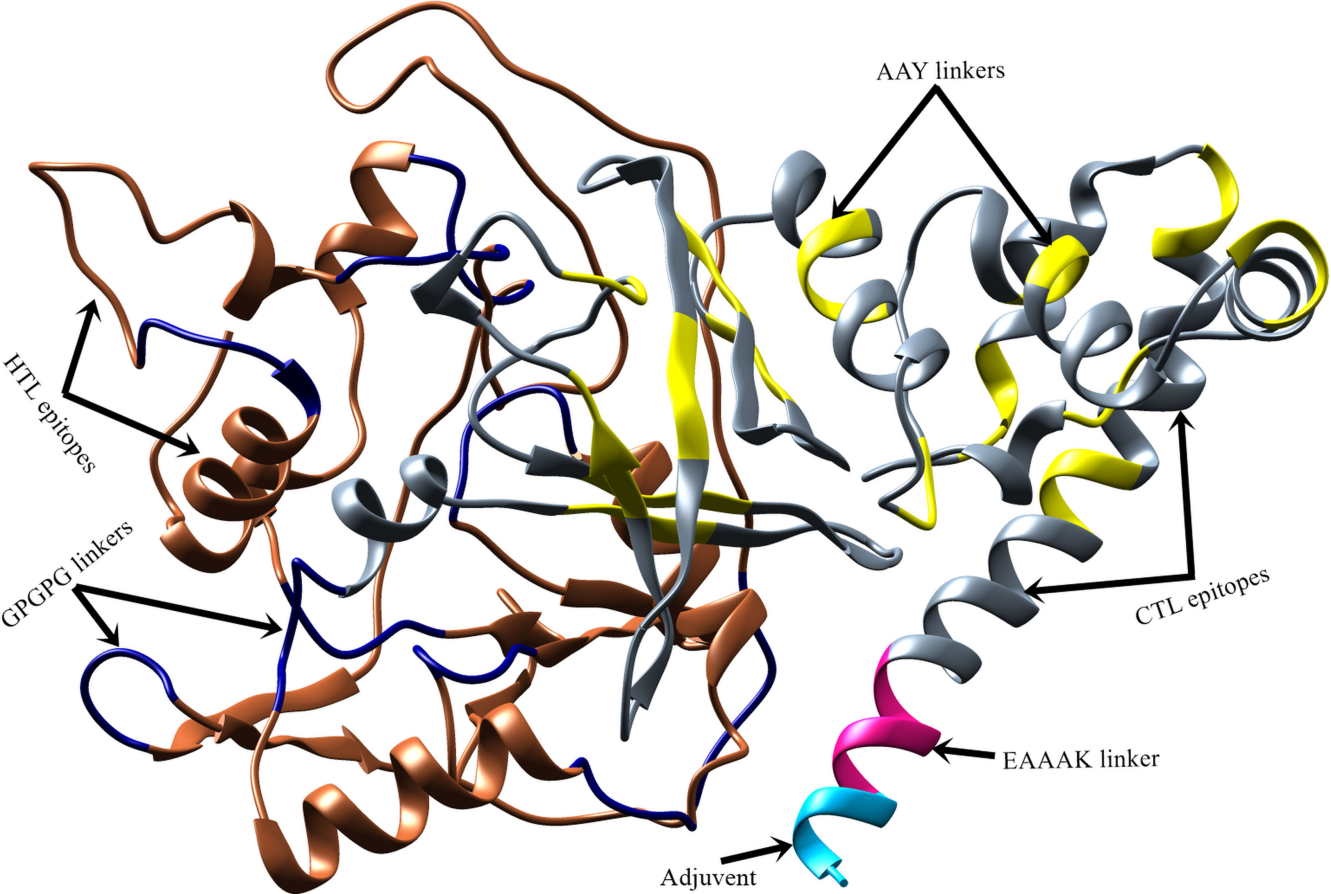
Predicted 3D structure of vaccine construct. Vaccine adjuvant is shown in cyan color, EAAAK linker in pink, CTL epitopes are in grey, AAY linkers and GPGPG linkers are in yellow and navy-blue color, respectively, while HTL epitopes are in sienna color.

The Robetta server (65) was employed to model the 3D structure of the vaccine (**Figure 4**). Robetta performed *ab initio* modeling to model the target sequence and generated five structures. Subsequently, model-1 was selected for additional analysis after a brief structural evaluation. Several computational tools were employed to validate the 3D structure of the vaccine construct. The ERRAT server (104) was used to predict the overall quality of the 3D structure with a quality score of 86.91, and ProSA-web server (68) was used to calculate the Z-score of the structure as -5.93.

### Structure Refinement

The accuracy of the predicted structure was improved using structure refinement tools (e.g., fragment-guided molecular dynamics algorithm). The evaluation of the refined vaccine structure on the Ramachandran plot showed that 88.4% of the residues were in most favored regions, 10.1% in additional allowed regions, 0.9% in generously allowed regions, and -0.6% in disallowed regions (**Supplementary Figure 2C**). In contrast, the unrefined structure had 42.6%, 40.7%, 11.9%, and 4.8% residues in most favored, additionally allowed, generously allowed, and disallowed regions, respectively.

### Disulfide Engineering of Vaccine Construct

Residues in the high-mobility region of the protein sequence were mutated with cysteine to perform disulfide engineering. Both inter- and intra-chain disulfide bonds were evaluated. A total of 49 pairs of amino acid residues with the capability to form disulfide bonds were predicted using the DbD2 server. After the evaluation of the pair residues in terms of chi3 and energy values, only two pairs were found potent for disulfide bond formation. Those residues were Ala92–Asp97 and Ala176 and Tyr190 (**Supplementary Figure 2B**). These residues were replaced with cysteine residues. The values of chi3 between -87 and +97 were considered for residue screening while the energy value was <2.5. The main objective of disulfide engineering was to make the vaccine construct less susceptible to host proteases by replacing susceptible residues with another one, e.g., cysteine, and to increase overall stability of the construct by introducing a disulfide bond (105, 106). The addition of the disulfide bond may alter the thermodynamic stability and disrupt the rate of folding and unfolding of the construct (106, 107). According to the Ramachandran plot (**Supplementary Figure 2**) of the construct, the introduction of the disulfide bond improved the stability of the construct by introducing conformational constraints to the backbone (108). The improved stability and removal of protease-susceptible residues may represent an improved immunogenicity by increasing the half-life of the construct in human body (108).

### Molecular Docking

**Figure 5:**
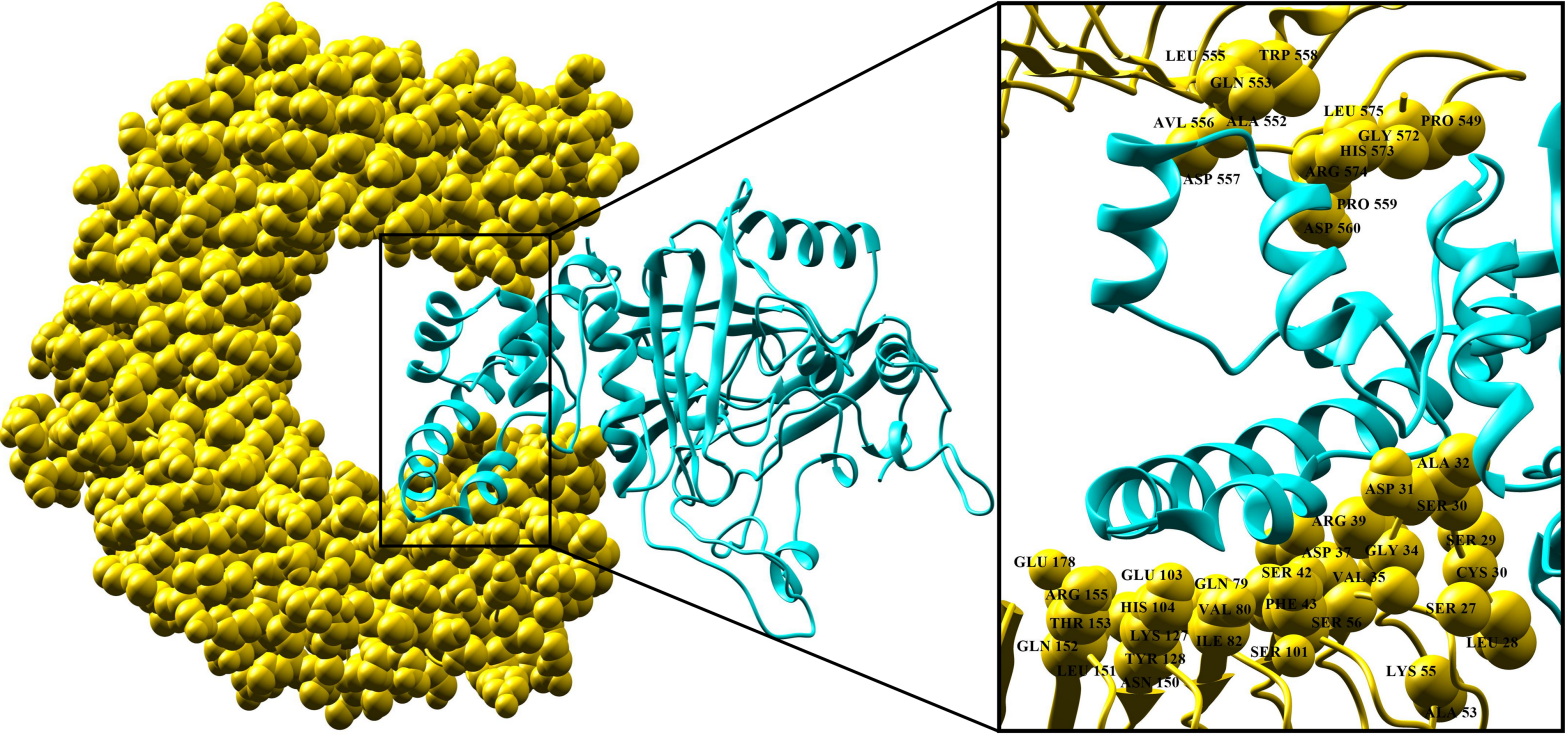
Docking pose of vaccine and TLR-2 receptor. TLR-2 receptor is in yellow and vaccine construct is in cyan. Interaction residues of the receptor are shown on the right-hand side.

The protein-peptide molecular docking technique was employed to predict the best binding mode of the vaccine construct to TLR2. As a membrane surface receptor, TLR2 is the most promiscuous TLR with respect to the pathogen-associated molecular pattern recognition derived from bacteria, virus, parasites, and fungi and its activation can result in the functioning of the intracellular signaling pathway of nuclear factor-kappa B and cytokine production leading to innate immunity activation (109). The PatchDock server (73) was used to generate the docked pose and electrostatic interactions between the vaccine construct and receptor (TLR2). Subsequently, the docked complex was further refined by using the FireDock tool (74). FireDock generated 10 docked solutions for the docked complexes with respective global energy values, and solution 9 of the docked complex with the lowest global energy was selected (**Figure 5**) (-29.77 kJ/mol global energy with TLR2, **Table 5**). The lowest global energy indicates the highest binding affinity between vaccines and receptors. Within 3Å of the vaccine construct Leu555, Trp558, Gln553, Leu575, Pro549, His573, Ala552, Asp557, Arg574, Pro559, Asp560, Ala32, Asp31, Ser30, Arg39, Asp37, Gly34, Ser29, Glu178, Glu103, Ser42, Ala53, Val35, Cys30, Arg155, His104, Val80, Phe43, Gln152, Lys127, Ile82, Tyr128, Ser101, Leu151, and Asn150 residues from TLR2 were involved in both hydrophilic and hydrophobic interactions. The selected docked complex was subjected to the molecular dynamics simulation for further stability analysis.

**Table 5:** Top ten best refined docked solutions generated using FireDock. Energy is presented in kJ/mol.

Rank	Patch Dock solution	Global energy	Attractive van der Waals	Repulsive van der Waals	Atomic contact energy	Hydrogen bond energy
1	9	-29.77	-35.11	16.48	2.74	-5.84
2	2	6.77	-3.42	0.29	1.12	-0.53
3	10	7.90	-42.29	28.94	15.35	-4.59
4	7	10.77	-2.66	0.00	0.61	0.00
5	8	12.95	-0.11	0.00	-0.26	0.00
6	3	17.06	-0.01	0.00	0.11	0.00
7	5	27.05	-7.14	28.20	9.40	0.00
8	4	134.23	-49.36	222.58	11.58	-4.62
9	6	738.14	-19.30	927.58	11.60	-5.66
10	1	6301.42	-63.00	7959.07	18.48	-15.44

### Molecular Dynamics Simulation

**Figure6:**
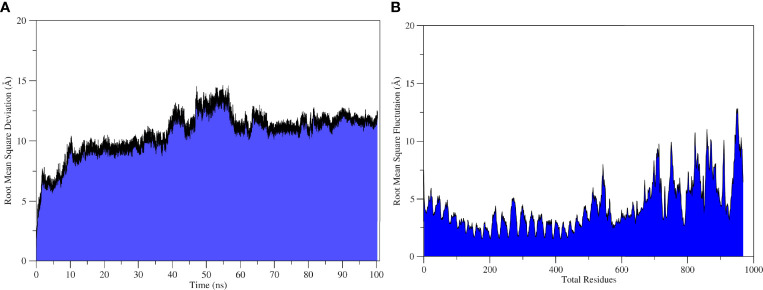
Statistical analysis of TLR2-Vaccine simulation trajectories. **(A)** Root Mean Square Deviation analysis, **(B)** root mean square fluctuation analysis.

The molecular dynamics simulation was performed to explore the physical movements at the atomic level and to confirm the stability of the docked complex. The root mean square deviation and root mean square fluctuation was determined to estimate the dynamic behavior and stability of the complex for the run of 100 ns. The root mean square deviation is the distance measure between backbone carbon alpha of superimposed proteins (110), and the graph showed an initial fluctuation of 7.6Å at 2 ns, increasing to 14Å (**Figure 6A**). The system reached equilibrium at 10–30 ns; some major fluctuations were observed at 30–64 ns; afterward, the system was re-equilibrated and remained stable until the end of the simulation run with an average root mean square deviation of 10.6 Å. Residual flexibility of the complex was analyzed *via* root mean square fluctuation (111) to understand the fluctuations of the residues and whether these variations affect the complex. **Figure 6B** revealed that residues of the vaccine construct (1–419) have mild fluctuations with a mean root mean square fluctuation of 3.08Å, which indicates the stability and uninterrupted interactions between the receptor and the construct. Major fluctuations with the highest peak of 12.5 Å were observed at C-terminal residues (700–976) (**Figure 6B**).

### MMPB/GB-SA Binding Free Energy

The MMPB/GB-SA methods are the most popular and preferred methods used to calculate the binding free energy in biomolecular studies such as protein-protein interactions. These methods are user-friendly, more accurate than docking/scoring methods, and considerably less expensive than the free energy perturbation method (112). The binding energies of the system understudies in both methods (MMPBSA/GBSA) are tabulated in **Table 6**. The system exhibited highly stable and very robust binding with a net binding energy of -43.27 kcal/mol and -68.99 kcal/mol in MMGBSA and MMPBSA, respectively. This energy was dominated by the gas-phase energy of -291.06 kcal/mol in both systems. Solvation was unfavorable as 247.79 kcal/mol in MMGBSA and 222.06 kcal/mol in MMPBSA. The gas-phase energy was mostly contributed by electrostatic energy (-162.74 kcal/mol) and less by van der Waals energy (-128.32 kcal/mol) in both methods.

**Table 6:** Free energy of binding, estimated by MMGBSA and MMPBSA for the complex.

MMGBSA			
TLR2-vaccine complex			
Energy component*	Average	Std. Dev	Std. Err. of Mean
VDWAALS	-128.32	12.23	1.38
EEL	-162.74	98.61	11.16
EGB	265.98	97.43	11.03
ESURF	- 18.19	1.85	0.20
DELTA G gas	-291.06	104.52	11.83
DELTA G solv	247.79	96.40	10.91
DELTA TOTAL	-43.272	13.37	1.51
**MMPBSA**			
**TLR2-vaccine complex**			
**Energy component**	**Average**	**Std. Dev**	**Std. Err. of Mean**
VDWAALS	-128.32	12.2324	1.38
EEL	-162.74	98.6140	11.16
EPB	237.64	99.7371	11.29
ENPOLAR	-15.57	1.2043	0.13
EDISPER	0	0	0
DELTA G gas	-291.06	104.5230	11.83
DELTA G solv	222.06	99.0910	11.21
DELTA TOTAL	-68.99	13.4570	1.52

*VDWAALS, van der Waals energy; EEL, electrostatic energy; EGB, polar solvation energy; ESURF, non-polar solvation energy; DELTA G gas, net gas phase energy; DELTA G solve, net solvation energy; DELTA TOTAL, total binding free energy of the system.

## Conclusions

We used immunoinformatic techniques in combination with subtractive proteomics to design a potent and safe multi-epitope vaccine that can be used against *P. gingivalis* infections. This study begins with the retrieval of the complete proteome of the pathogen and removal of irrelevant proteins to determine the most suitable protein targets composed of non-allergic, antigenic, virulent, and high-affinity binders with DRB*0101 multi-epitope peptides. Predicted epitopes showed higher worldwide human population coverage of 99.93%. Immunoinformatic tools and online web servers were used to predict B and T cells from the selected proteins. CTL and HTL epitopes were fused using appropriate linkers. Molecular docking showed the strong binding affinity of the vaccine construct to the innate immune receptor (TLR2), thus allowing adaptive immunity and prompt response against the pathogen. The molecular dynamics simulations illustrated the highly stable molecular interactions and predicted the binding mode of the construct. Further, binding free energy calculations support the docking and simulation findings as a highly stable nature of the complex. The designed vaccine construct was immunogenic in our *in silico* evaluation; nevertheless, the extent to which it could prevent *P. gingivalis* infection is unknown. Immunoinformatics approaches have been successfully applied to a number of pathogens to screen for protective antigens, such as the *Meningococcus* B (*Men*B) vaccine developed recently. Similarly, *Chlamydia*, *Staphylococcus aureus*, and *group* A *Streptococcus* are successfully addressed using such *in silico* methods. The vaccine construct developed in this study is ready to be evaluated in experimental studies to disclose its immunogenicity against the *P. gingivalis* pathogen.

## Data Availability Statement

The original contributions presented in the study are included in the article/**Supplementary Material**. Further inquiries can be directed to the corresponding authors.

## Author Contributions

BS: project design, investigation, data curation, original draft preparation, figure preparation, and referencing. SA: Figure preparation, reference management, reviewing, and editing. JS: Figure preparation, reference management, reviewing, and editing. HWK: Supervision, conceptualization, methodology, and writing—reviewing and editing. DN: Supervision, conceptualization, methodology, patient’s sampling acquisition, writing—reviewing and editing, and project management. All authors contributed to the article and approved the submitted version.

## Funding

This research was supported by a grant of the Korea Health Technology R&D Project through the Korea Health Industry Development Institute (KHIDI), funded by the Ministry of Health and Welfare, Republic of Korea (grant number: HI21C1659). This research was also supported by the Chung-Ang University Research Grants, 2020.

## Conflict of Interest

The authors declare that the research was conducted in the absence of any commercial or financial relationships that could be construed as a potential conflict of interest.

## Publisher’s Note

All claims expressed in this article are solely those of the authors and do not necessarily represent those of their affiliated organizations, or those of the publisher, the editors and the reviewers. Any product that may be evaluated in this article, or claim that may be made by its manufacturer, is not guaranteed or endorsed by the publisher.

## Abbreviations

MHC, Major histocompatibility complex; TLR, toll-like receptor; HTL, helper T lymphocytes; CTL, cytotoxic T lymphocyte.
