# Supplementary material for: Computational Design of a Multi-Epitope Vaccine Against Porphyromonas gingivalis
Source: Front Immunol. 2022 Feb 18;13:806825. doi: 10.3389/fimmu.2022.806825 (PMC8894597; doi:10.3389/fimmu.2022.806825)
Supplement: Supplementary file 1 [file DataSheet_1.docx]

**Supplementary files**


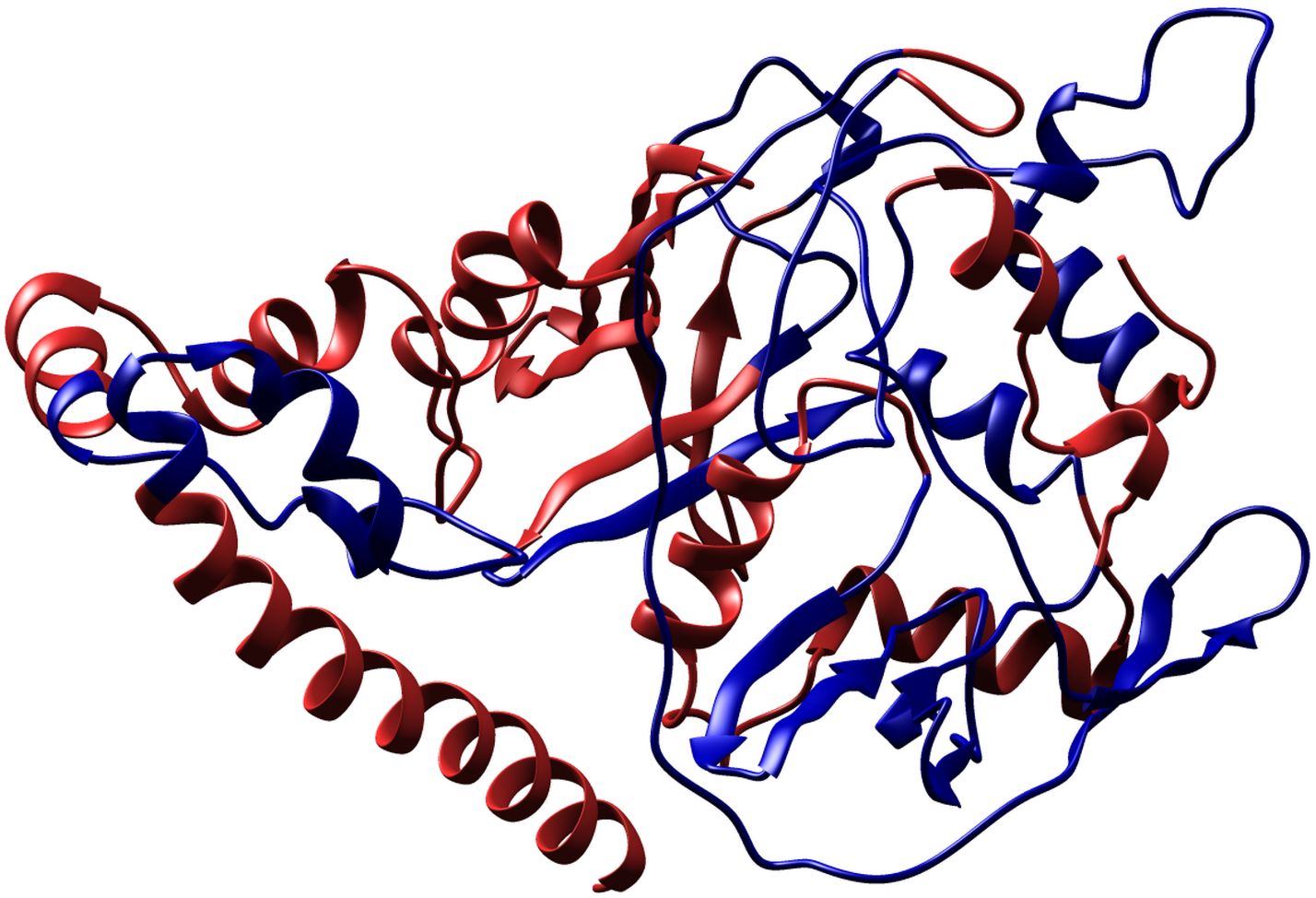


**Supplementary Figure 1. Graphical depiction of B-cell epitopes.** Linear B-cell epitopes are represented in Navy-blue.


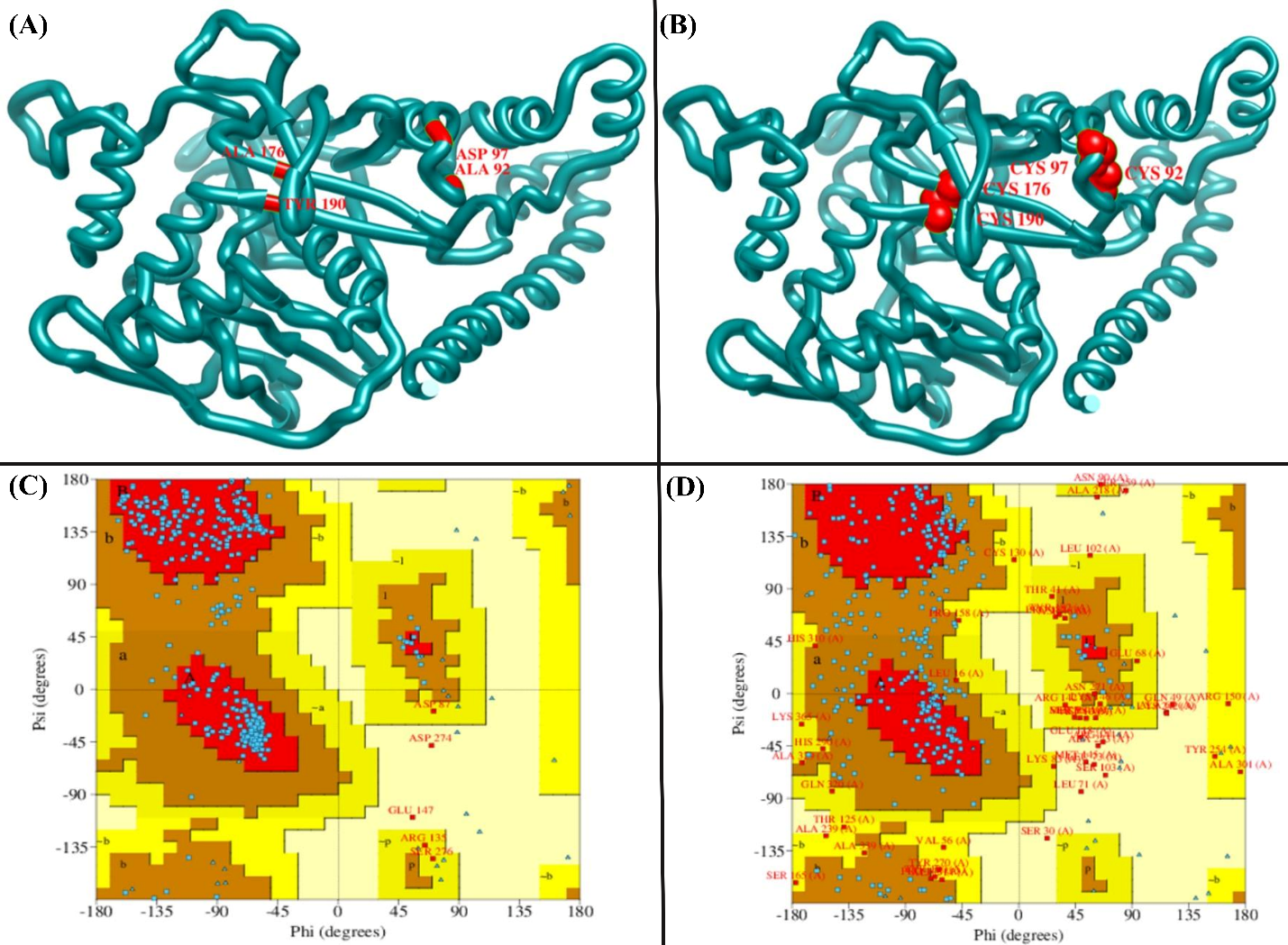


**Supplementary** **Figure 2.** **Representation of refined and disulfide engineered 3D structures of vaccine construct**. The refined 3D structure of the vaccine is presented in (A) and disulfide engineered structure in (B), while the Ramachandran plot of refined and disulfide engineered structure is illustrated in (C) and (D), respectively.

**Supplementary Table 1.** Worldwide human population coverage by the predicted epitopes

| **Population** | **Coverage (%)^a^** | **Average hit^b^** | **PC90^c^** |
| --- | --- | --- | --- |
| East Asia | 41.1% | 1.11 | 0.17 |
| China | 43.15% | 1.19 | 0.18 |
| Europe | 99.95% | 10.21 | 6.64 |
| North America | 99.95% | 8.81 | 6.33 |
| South Asia | 52.58% | 2.02 | 0.21 |
| Southwest Asia | 59.28% | 2.41 | 0.25 |
| Central Africa | 46.69% | 1.07 | 0.19 |

^a^ Projected population coverage
^b^ Average number of epitopes hits / HLA combinations recognized by the population
^c^ Minimum number of epitope hits / HLA combinations recognized by 90% of the population

**Supplementary Table 2.** Predicted discontinuous B-cell epitopes

| **Residues** | **Contact number** | **Score** |
| --- | --- | --- |
| Ser, Lys, Lys, Lys, Lys, Glu, Ala, Ala, Ala | 11, 6, 11, 3, 4, 20, 18, 5, 10, | 5.59, 5.78, 4.72, 4.51, 4.75, 1.85, -1.29, -0.13, -0.14 |
| Met | 6 | -3.23 |
| Glu, Asn | 9, 10 | -3.12, -3.60 |
| Asn, Ala, Asn, Gly, Glu, Lys | 10 ,18, 0, 3, 4, 9 | -2.01, -3.19, -1.14, -2.24, -1.32, -3.43 |
| Glu | 16 | -3.56 |
| Ala, Lys, Glu, Tyr, Gly, Pro, Gly, Pro, Gly, Arg | 23, 22, 21, 23, 22, 23, 31, 25, 23, 17 | -1.53, 0.61, 0.33, 0.67, 2.61, 3.32, 2.02, 2.69, 4.60, 5.36 |
| Lys, Gly, Lys, Ile, Arg | 12, 5, 0, 17, 9 | 5.79, 4.27, 3.65, 4.27, 5.89 |
| Thr, Tyr, Ile, Asn, Leu, Leu, Leu, Thr, Met, Gly | 2, 12, 23, 9, 8, 13, 15, 9, 12, 17 | 5.12, 3.73, 1.76, 2.37, 1.29, 0.18, -2.33, -2.10, -1.10, -2.90 |
| Pro, Gly, Pro, Gly, Asn, Arg, Lys, Gly | 11, 2, 0, 6, 7, 13, 19, 23 | 0055, 3.85, 3.77, 3.78, 1.49, -1.23, -0.18, -2.31 |
| Gln | 27 | -3.26 |
| Gly, Val, Gln, Tyr, Arg, Ala, Asp, Lys, Ser, Tyr | 27, 25, 22, 26, 8, 18, 10, 2, 19, 12 | -3.61, -3.37, -1.14, -0.69, 0.76, -0.61, 0.93, 1.77, -0.26, 0.53 |
| Ile, Leu, Ala, Gly, Pro | 20, 25, 26, 29, 28 | 0.27, -0.52, -0.50, -1.75, -1.31 |
| Gly, Pro, Gly, Ser, Gln, Ser, Gly, Val, Gln, Tyr | 21, 25, 26, 28, 12, 22, 18, 30, 17, 17 | 1.15, 1.12, 1.65, 0.60, 2.63, 1.01, 1.27, -0.611, -0.03, 0.58 |
| Arg, Ala, Asp, Lys, Ser | 16, 0, 9, 19, 22 | -0.33, 0.42, -0.39, -0.26, -1.20 |
| Tyr, Ile, Leu, Gly, Pro, Gly, Pro, Gly, Lys, Asn | 30, 17, 26, 26, 24, 25, 29, 30, 31, 19 | -1.05, 0.59, -1.10, 0.45, 2.19, 2.15, 2.39, 2.58, 1.16, 3.43 |
| Tyr, Arg, Leu, Ala | 4, 18, 28, 17 | 4.04, 2.24, -0.01, 0.97 |
| Asn, Phe, Ser, Thr, Arg, Phr, Leu, Ser, Gly, Pro, Gly, Pro | 5, 24, 7, 3, 22, 13, 5, 13, 18, 16, 22, 20 | -0.01, -1.77, 1.14, 0.99, -1.30, 0.22, 0.22, -0.42, -0.66, -1.77, -2.14, -3.58 |
| Thr, Ala, Lys, Asp, Pro, Phe, Arg, Val, Ser, Ala | 12, 2, 20, 10, 0, 24, 9, 16, 15, 27 | -1.34, -0.10, -1.67, -114, 1.17, -2.08, 0.02, -0.56, -0.52, -0.56 |
| Ser, Ala, Arg, Tyr, Asp, Gly, Pro, Gly, Pro, Gly | 2, 26, 2, 15, 18, 10, 0, 2, 11, 5 | 2.98, 2.18, 5.81, 5.59, 5.18, 5.76, 6.43, 7.15, 7.24, 6.78 |
| Asn, Val, Val, Tyr, Phe, Arg, Ile, Asn, Ser, Ala | 11, 13, 28, 6, 31, 6, 31, 6, 21, 15 | 6.08, 5.30, 2.29, 3.82, 0.60, 2.37, -1.29, -0.71, -0.65, 0.31 |
| Lys, Ile, Asp, Arg, Asn, Gly, Pro, Gly, Pro | 0, 17, 29, 5, 3, 15, 23, 14, 16 | 4.38, 2.44, 2.71, 6.26, 7.75, 4.05, 1.77, 0.86, -3.09 |
| Asn | 3 | -3.43 |
| Ala, Lys, Ile, Asp, Arg, Gly, Pro, Gly, Pro, Gly, Leu, Ile, Arg | 13, 4, 21, 0, 0, 15, 12, 20, 3, 0, 22, 19, 21 | -1.07, 1.26, -0.80, 1.26, 1.10, -0.82, -1.58, -1.58, 0.08, 0.96, -1.14, -3.09, -3.14 |
| Leu | 7 | -2.44 |
| Pro | 10 | -1.42 |
| Gly, Pro, Gly, Glu, Leu, Ile, Arg, Ile, Leu, Thr | 10, 13, 9, 26, 8, 21, 16, 6, 12,15 | -3.03, -1.71, 1.06, 0.87, 2.18, 0.47, 0.83, 1.80, 0.31, 0.05 |
| Asp, Asn, Pro, Asp, Ile, Arg, Ile, Glu, Gly, Pro | 4, 11, 0, 0, 15, 7, 20, 22, 9, 4 | 1.16, 0.72, 1.37, 4.24, 0.15, 1.50, 0.72, 1.40, 1.73, 2.53 |
| Gly, Pro, Gly, Asp, Glu, Ile, Ile, Arg, Ile, Leu | 13, 10, 22, 6, 12, 22, 17, 7, 17, 27 | 1.92, 1.75, 0.64, 3.48, 4.20, 3.16, 2.93, 5.04, 5.37, 4.28 |
| Thr, Asp, Asn, Pro, Asp, Ile, Arg, Ile | 19, 10, 15, 22, 5, 21, 13, 19 | 5.50, 7.16, 5.84, 5.10, 6.13, 3.41, 3.98, 1.70 |
